# Cranial Nerve Foramina: Part II – A Review of the Anatomy and Pathology of Cranial Nerve Foramina of the Posterior Cranial Fossa

**DOI:** 10.7759/cureus.2500

**Published:** 2018-04-18

**Authors:** Bryan Edwards, Joy MH Wang, Joe Iwanaga, Marios Loukas, R. Shane Tubbs

**Affiliations:** 1 Department of Anatomical Sciences, St. George's University School of Medicine, St. George, GRD; 2 Seattle Science Foundation; 3 Neurosurgery, Seattle Science Foundation

**Keywords:** foramen, posterior fossa, cranial nerve, foramen magnum, hypoglossal canal, jugular foramen, internal acoustic meatus, skull base

## Abstract

Cranial nerve foramina are integral exits from the confines of the skull. Despite their significance in cranial nerve pathologies, there has been no comprehensive anatomical review of these structures. Owing to the extensive nature of this topic we have divided our review into two parts; Part II, presented here, focuses on the foramina of the posterior cranial fossa and discusses each foramen’s shape, orientation, size, surrounding structures, and structures that pass through it. Furthermore, by comparing foramen sizes against the cross-sectional areas of their contents, we determine the amount of free space available within each. We also review lesions that can obstruct each foramen and discuss the clinical consequences.

## Introduction and background

Cranial nerve foramina are integral exits from the confines of the skull. On their long intracranial journeys and subsequent passage through these skeletal portals, cranial nerves can travel alone or with accompanying vascular structures. The foramina can sometimes be too small or pathological obstructions (e.g., achondroplasia, fibrous dysplasia, osteopetrosis) can develop and impinge upon those structures, with potentially severe clinical consequences.

**Figure 1:**
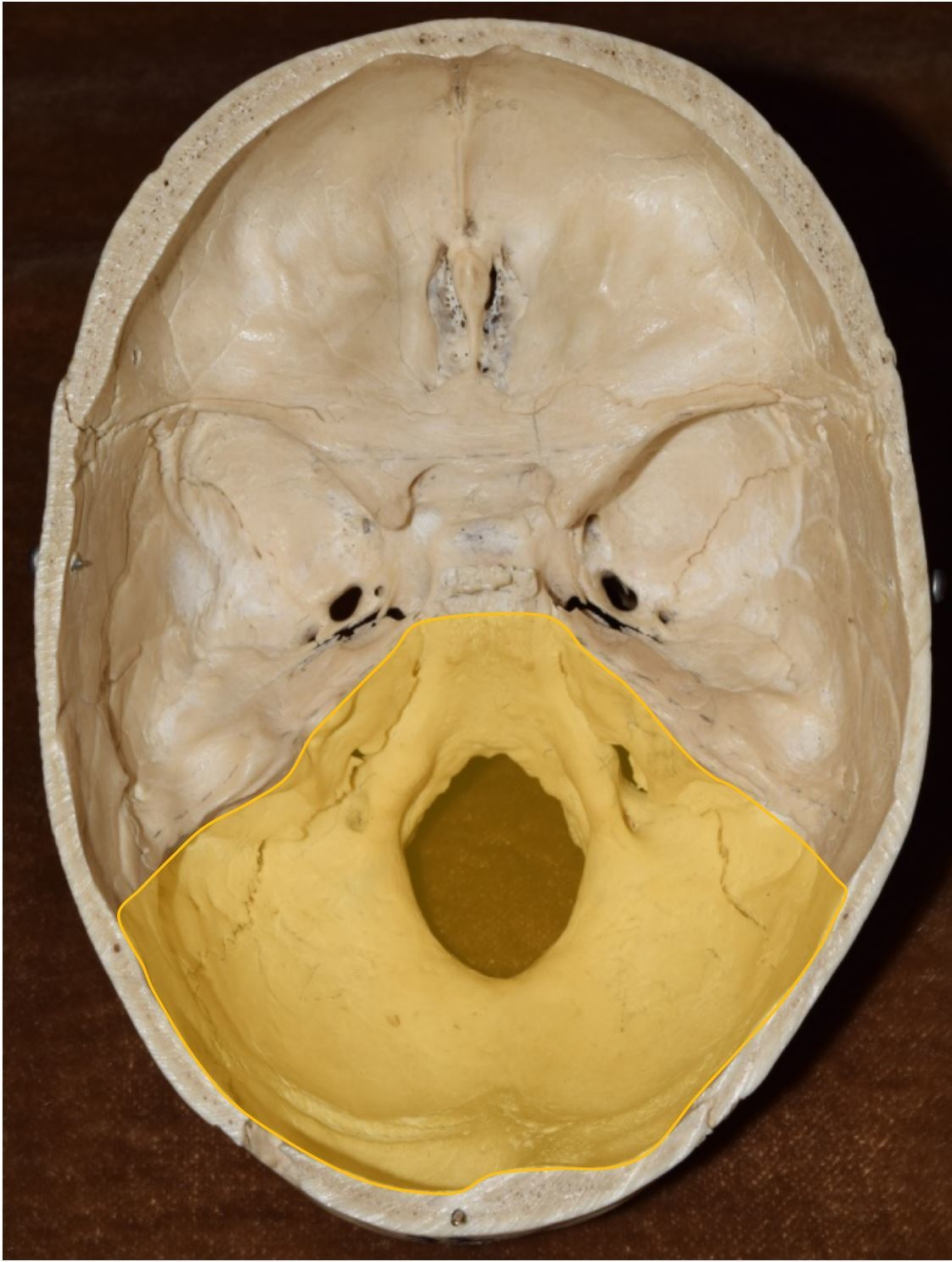
Superior view of cranial floor. Yellow - Posterior cranial fossa

In this review, we describe the anatomy of the cranial nerve foramina of the posterior cranial fossa (highlighted in yellow in Figure 1) in terms of locations within the skull, shapes, dimensions, crucial surrounding structures, and documented variations. The structures passing through these foramina and their corresponding sizes are also reviewed by comparing their respective cross-sectional areas. Finally, pathological obstructions of the foramina and impingement on their contents are reviewed, along with the corresponding clinical consequences. To our knowledge, this is the first comprehensive review of the cranial nerve foramina of the posterior cranial fossa.

Limitations

Information regarding structural diameters, sizes of lesions, and measurements of masses extending into the foramina is seldom or never reported in the literature.

## Review

Posterior cranial fossa

Foramen Magnum (FM)

**Figure 2:**
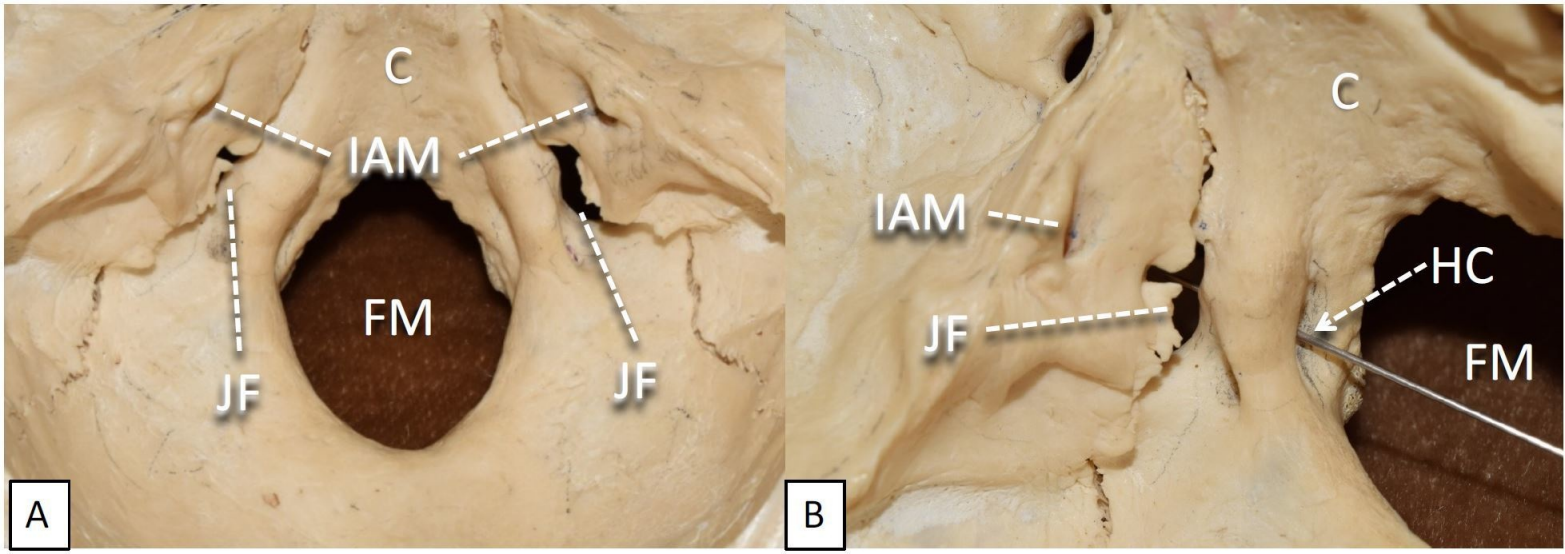
Close up view of cranial nerve foramina within posterior cranial fossa. (A) Superior view. (B) Oblique view C: Clivus; IAM: Internal acoustic meatus; JF: Jugular foramen; HC: Hypoglossal canal; FM: Foramen magnum.

Lying at the base of the skull, the final point of departure for nerves, vessels, and other structures, the foramen magnum (Figure 2) is a large, oval opening lying perfectly flat in the horizontal plane. Completely contained within the occipital bone, its borders are formed anteriorly by the inferior aspect of the downward-sloping clivus, laterally on both sides by the jugular tubercles, and posteriorly by the edge of the squamous part of the occipital bone. Immediately adjacent to the lateral edges of the FM, on the exocranial surface of the occipital bone, are the occipital condyles. With an average area of 826.44 mm^2^, the FM is the largest gateway in and out of the cranial cavity. Furthermore, it is one of the few foramina for which there is a significant size difference between the sexes, the average cross-sectional area being more than 100 mm^2^ smaller in females [1].

Important structures surround the FM on the intracranial and extracranial surfaces. Lateral to it, roughly 10 mm away, is the hypoglossal canal [1]. Superior to it are the cerebellum and brain stem; rootlets for cranial nerves (CN) IX, X, and XI emerge at this level. Running through the FM are the medulla oblongata with its covering meninges, the V4 segment of the vertebral arteries, and the spinal roots of the accessory nerve [2]. Inferiorly, the spinal cord and its meninges continue caudally beyond the cranium. Obstructions in this foramen, either from lesions within the FM itself or from lesions of surrounding structures, can have serious consequences. Major symptoms include fainting and lightheadedness from compression and narrowing of the vertebral arteries, respiratory and autonomic dysfunctions from compression of the medulla, motor dysfunctions, and head and neck pain from meningeal irritation.

Intrinsically, meningiomas are the most common FM tumors, representing 1.8–3.2% of all intracranial meningiomas [3]. Mahore et al. reported a patient with a large anterolateral mass measuring 3.1 x 2.7 x 2.9 cm who suffered from hyperreflexia of all four limbs and “drop attacks” – sudden episodes of falling without losing consciousness [4]. Once the lesion was excised, the symptoms resolved. Tsao et al. reported on a patient with meningothelial meningioma in the FM who suffered from a myriad of symptoms including progressive dysphagia with coughing when trying to swallow; weight loss; numbness, tingling, and pain over the right side of the face; absent gag reflex; increased jaw jerk reflex; positive Babinski sign; and gait instability. Videofluoroscopy revealed epiglottis retroflexion [5]. Also, the foramen reportedly becomes stenosed secondary to achondroplasia, a genetic condition arising from an autosomal dominant mutation of the fibroblast growth factor gene [6]. The result is most often a thickened opisthion encroaching upon the foramen, leading to a wide range of symptoms. Achondroplasia respiratory difficulty syndrome can result from compression of the respiratory centers in the brain stem, causing sleep apnea and respiratory failure [6-8]. Furthermore, hydrocephalus can result from CSF blockage. Arishima et al. reported two similar cases of children suffering from myelopathy, numbness in hands and feet, hyperreflexia, and spastic gait, later confirmed on magnetic resonance imaging (MRI) to have opisthion overgrowth. Arachnoid cysts over the brain stem within the FM can compress the structures running through it, causing syringomyelia. Huang et al. reported a 5 cm cystic lesion in the posterior FM of a 43-year-old man with weakness, numbness, loss of urinary and bowel control, wasting of upper limb muscles, and decreased tendon, cremaster, and anal reflexes. Intracranial dural arteriovenous fistulas have also been reported with the occipital and ascending pharyngeal arteries, causing obstruction of the FM and resulting in symptoms such as progressive paraparesis, urinary retention, loss of consciousness, absent reflexes, and lack of sensation at the T4 level [9]. Metastatic prostate cancer has also been shown to cause compression at this site. In one case, a 73-year-old man developed weakness, gait dysfunction, falls, right facial hemiparesis, and hyperreflexia from a 2.0 x 1.4 x 1.2 cm metastatic mass displacing the spinal cord within the FM. The symptoms improved after excision [10]. Osteochondroma, a benign bone tumor, has also been found in the FM, arising from the occipital bone itself and compressing the medulla. This caused limb weakness, pain at the back of the head, spastic quadriparesis, hyperreflexia, and positive Hoffman’s sign and extensor plantar response, all of which resolved upon surgical excision of the tumor [11].

Extrinsic lesions can also compress structures within the FM. Chiari malformations type 1 have commonly been associated with a stenosed or malformed FM. In Chiari malformation type 1, the cerebellar tonsils herniate through the foramen. This can result in brain stem/spinal cord compression or interfere with the flow of CSF, potentially resulting in syringomyelia. Within the posterior cranial fossa, masses above the cerebellum can push down on the cerebellar tonsils and act as a pseudo-Chiari type 1 malformation. Lesions such as cerebellar vermis medulloblastoma, dermoid tumor, astrocytoma of the posterior cranial fossa, and meningioma of the cerebellar tentorium have all presented in this manner [12].

Hypoglossal Canal (HC)

The hypoglossal canal (Figure 2) is located on the anterior aspect of the occipital bone, inferolateral to the inferior edge of the clivus and 8 mm inferomedial to the jugular foramen [13]. From a superior view of the intracranial floor from the front, these bilateral cylindrical canals are located at approximately 2 o’clock and 10 o’clock from the FM. They travel approximately 7.8 mm diagonally outwards in the base of the skull and open immediately lateral to the occipital condyles. The canal can sometimes be separated into two compartments by an ossified septum. Prevalence is reported as 20–30%, most occurring unilaterally [14-15]; bilateral septated canals are rare, occurring in less than 5% [15]. Also, incomplete bony spurs rather than a complete septum traverse the canal opening in 13–26% of cases. Intracranially, the foramen has an average area of 5.51 x 4.25 mm, whereas the extracranial opening is narrower, at 4.66 x 3.21 mm; however, variations of up to two millimeters in each dimension have been documented [15]. Within the foramen, the canal length is typically in the 7–9 mm range [13]. The cerebellum, brain stem, and carotid and vertebral arteries lie superior to the HC, as to the FM, while the body of the first cervical vertebra and the spinal cord are inferior [16].

Running through the foramen, as the name implies, is the hypoglossal venous plexus, and in 45% of cases the meningeal branch of the ascending pharyngeal artery [17]. Using the typical diameter of the hypoglossal nerve (1.64–1.70 mm) and the formula for the area of an ellipse (A = πrarb), we infer that the cross-sectional area of the nerve is 2.11–2.27 mm^2^ [17]. Since the cross-sectional area of the endocranial foramen, the narrowest portion of the HC, is 11.75 mm^2^, there is a difference of over 7 mm^2^ between these two structures. Assuming the area of the accompanying vessels to be small, there is ample space for these structures within the canal.

Despite the extra space, obstructions within the HC do occur, most commonly secondary to extrinsic lesions of surrounding structures. If there is impingement on the hypoglossal nerve we expect signs and symptoms largely affecting the tongue such as atrophy, fasciculation, and deviations, which can subsequently result in dysphagia. Different presentations are possible depending on the size and location of the lesion. The most common tumors affecting the HC arise from the area of the jugular foramen [18]. One example is a left jugulotympanic paraganglioma with extensions into the middle ear and partially into the hypoglossal foramen. The patient presented with hearing loss and vocal cord paralysis but, interestingly, without tongue problems. Calzada et al. reported a case of an intradural meningioma located in front of the brain stem and extending into the HC, causing left-sided tongue atrophy, dizziness, and disequilibrium. Schwannomas of the hypoglossal nerve represent 5% of all non-acoustic schwannomas and have been known to extend into the canal; 85.75% of such patients present with hypoglossal nerve palsy [18]. In one case, a 61-year-old patient with a right-sided parapharyngeal mass adjacent to the extracranial foramen of the HC exhibited ipsilateral tongue deviation, fasciculation, and atrophy, with slight ptosis and neck pain. Cystic schwannomas of the hypoglossal nerve can also extend into the canal. In one case, a 4.7 x 3.8 x 2.9 cm cystic schwannoma originating from the level of the FM extended through the length of the canal, causing destruction of the right HC and compressing the right cerebellum and brain stem along the way. The patient had right tongue deviation, atrophy, and fasciculation, along with vomiting, vertigo, gait unsteadiness, and right-sided headaches. Neurinomas of the hypoglossal nerve extending from the level of the FM can also extend into the canal causing a similar presentation [18].

Jugular Foramen (JF)

On the crux between the occipital bone and the petrous part of the temporal bone, the Jugular Foramen (Figure 2) is superomedial to the HC, the jugular tubercle separating the two structures [19,20], and inferoposterior to the internal acoustic meatus. The anterolateral border is the petrous part of the temporal bone while the inferior border is the occipital bone. The JF is directly lateral to the carotid canal. Its shape, loosely resembling the letter U, is irregular and asymmetrical; this has been attributed to the development of dural sinuses in the area [21,22]. As in the HC, bony processes originating from the temporal and/or occipital bone can separate the foramen into two compartments: an anterior compartment known as the pars nervosa and a posterior compartment known as the pars vascularis [20,21]. The prevalence of compartmentalization of the JF ranges from 1% to 13% unilaterally [22] and is 4% bilaterally [23]. There are also reports of the JF being divided into three compartments [24]. In 75% of the population, the right JF is larger than its left counterpart [20]. The dimensions of both foramina can vary [21], average dimensions on the right and left being 23.62 x 7.83 mm and 22.86 x 6.83 mm, respectively [25].

Several important structures surround the JF and knowledge of their spatial relationships is key to understanding the potential clinical effects of nearby lesions. Anterosuperiorly is the inner ear complex, continuing from the internal acoustic meatus located superior to the JF. The mastoid process and parotid gland are located on the lateral aspect extracranially. Moving medially from the mastoid process, sandwiched between it and the JF, is the external opening of the stylomastoid foramen from which the facial nerve exits. Intracranially, the tentorium cerebella lies above the JF, and inferiorly is the parapharyngeal space [26]. On the exocranial, anterior edge of the JF is part of the attachment site of the prevertebral fascia, which also attaches laterally to the mastoid. The prevertebral fascia contains the vertebral column and surrounding musculature [27].

The structures passing through the JF include CN IX, X, and XI, Jacobsen’s nerve, Arnold’s nerve, the meningeal branches of the ascending pharyngeal and occipital arteries [24], the internal jugular vein, and the inferior petrosal sinus. The pars nervosa contains the inferior petrosal sinus [21], the glossopharyngeal nerve, and Jacobsen’s nerve, while the pars vascularis contains the internal jugular vein, CN X, XI, and the meningeal branches of the ascending pharyngeal and the occipital artery [21,22]. The diameters of CN IX, X, and XI were measured as 2.3 mm, 4.6 mm, and 3.5 mm, respectively. Using the formula for the area of a circle and summing these cranial nerve areas, the total is 30.39 mm^2^. The cross-sectional area of the internal jugular bulb was measured as 160 mm^2^ [28]. Comparing this with the total cross-sectional area of the jugular foramen – 584.36 mm^2^ on the right, 493.30 mm^2^ on the left – there is over 400 mm^2^ and 300 mm^2^ of free space on the right and left, respectively. Intrusion into this free space, leading to obstruction and impingement on the structures within, can result in several clinical presentations.

Vernet’s syndrome, also known as jugular foramen syndrome, is caused by compression of the three main cranial nerves running through the JF, namely IX, X, and XI. Compression of these nerves is expected to cause loss of taste and sensation in the posterior third of the tongue, absent gag reflex, hoarseness, vocal pitch changes, dysphagia, and weakness of the ipsilateral trapezius and sternocleidomastoid. The most common causes of Vernet’s syndrome are meningiomas within the foramen [29], proposed to arise from arachnoid-lining cells surrounding the jugular bulb [26]. Paragangliomas, otherwise known as glomus jugular tumors, are also a common cause of JF obstruction. These tumors tend to grow along the path of least resistance, causing extensive invasion of surrounding structures such as the mastoid air cells, inner ear structures, and vascular channels [29]. Internal structures running through the foramen can also enlarge to cause obstruction. The internal jugular vein has been reported to cause Vernet’s syndrome when dilated to 288 mm^2^ [30]. Schwannomas of CN IX, X, or XI represent 3–4% of all intracranial schwannomas and can produce classical Vernet’s syndrome symptoms; their extension into the inner ear and/or compression of the cerebellum can also cause tinnitus, deafness, vertigo, and ataxia [26,29]. Recurrent and chronic otitis media causing osteomyelitis and abscesses of the surrounding bony structures can lead to narrowing of the JF and subsequent impingement on the structures within. Infection by Varicella zoster causes inflammation of the vagus nerve at the level of the JF, its swelling subsequently impinging on the other nerves within the foramen. Kawabe et al. reported a case of Vernet’s syndrome who presented with dysphagia, hoarseness, neck pain, and fever; subsequent MRI with CSF analysis confirmed a lesion around the vagus nerve within the JF secondary to an active Varicella infection [31]. The patient’s symptoms resolved quickly after the virus was treated. The meningeal branch of the ascending pharyngeal artery is susceptible to inflammatory changes due to giant cell arteritis; this has also been reported to cause Vernet’s syndrome in two cases. Although dysphagia, hoarseness, and dysphonia were reported in these cases, the inflammation secondary to vasculitis did not seem to affect the spinal accessory nerve [32]. This could be because of the anatomical course of these structures, as the spinal accessory nerve is typically more lateral to the artery than the vagus and glossopharyngeal nerves.

Metastatic cancers can represent up to 36% of lesions affecting the foramen, most arising from the breast, lung, or kidney. If a patient with confirmed diagnosis of such a cancer begins to experience craniofacial pain, it is highly suggestive of cranial base metastasis, so further imaging is indicated. In addition to symptoms of Vernet’s syndrome, patients can also experience headaches, mastoid pain, ear pain, vertigo, vomiting, gait instability, and signs of Horner’s syndrome [24]. Hayward et al. reported a case of non-small cell adenocarcinoma of the right upper lobe that spread to involve the petrous apex, jugular bulb, cochlear aqueduct, occipital condyle and hypoglossal canal [24]. Lymphomas, melanomas, and squamous cell carcinomas of the face and oral cavity have been shown to undergo retrograde perineural spread, invading areas within and/or surrounding the JF [29]. In children, rhabdomyosarcoma of the nasopharynx and masticator space can infiltrate the skull base at the level of the JF, invading the surrounding tissue. In one patient, a clear cell carcinoma of the parotid extended medially to reach and enter the JF; however, this patient luckily presented with nothing more than a bulge in the oropharynx and headache. Chordomas and chondrosarcomas have also been reported and tend to present with progressive CN symptoms in people between 20 and 40 years of age.

Given the close proximity of the entrance of the hypoglossal canal, space-occupying lesions from the JF can extend to impinge on the hypoglossal nerve. In Villaret’s syndrome, there is compression of CN IX-XII with signs of sympathetic involvement. Although lesions causing Villaret’s typically involve the retroparotid space where the cranial nerves from the JF meet the hypoglossal nerve [33], case reports have demonstrated that in extremely rare circumstances lesions such as schwannomas and jugular phlebectasia can extend from the JF and have hypoglossal involvement [34].

Internal Acoustic Meatus (IAM)

At the upper reaches of the posterior cranial fossa, the IAM (Figure 2) is the most superior foramen, located completely within the petrous part of the temporal bone. It is lateral from the superior aspect of the clivus, superior to the JF, and inferior to the groove of the superior petrosal sinus. It is oval, with average dimensions 4.73 x 3.78 mm, over 90% of skulls having the same size bilaterally [15]. Superior to the meatus is the tentorium cerebelli, while the cerebellum and brain stem are posteroinferior. Laterally, the meatus opens to the structures of the inner ear.

Running through the meatus are the intracranial segments of the facial nerve, the vestibulocochlear nerve, and the labyrinthine artery – a branch of either the anterior inferior cerebellar or the basilar artery. Nakamichi et al. reported the cross-sectional areas of the cochlear and facial nerves to be 10.7 mm^2^ and 0.83 mm^2^, respectively [35]. Using the reported diameter of 0.19 mm for the labyrinthine artery [36], we can use the formula for the area of a circle to determine a total cross-sectional area of 0.028 mm^2^. Summing the cross-sectional areas of all the structures gives 11.56 mm^2^. Using the formula for the area of an ellipse, we can take the dimensions of the meatus and calculate the cross-sectional area as 14.04 mm^2^. This implies less than 3 mm^2^ of free space between the walls of the foramen and the internal structures, significantly less than in the FM or JF. Given the limited free space available we would expect smaller lesions to produce significant clinical consequences.

With compression of the vestibulocochlear nerve we expect symptoms of hearing loss, tinnitus, and imbalance, whereas with compression of the facial nerve we expect impairment of facial muscles, and facial pain. Most lesions affecting the IAM and its structures arise intrinsically. Osteomas, most commonly found in the external auditory canal and the mastoid process, rarely involve the internal auditory canal so they seldom cause CN VII and VIII symptoms [37]. However, Suzuki et al. reported osteomas within the internal canal in a patient presenting with vertigo, tinnitus, sensorineural hearing loss and imbalance [37]. Estrem et al. also reported osteomas growing from the posterosuperior lip of the internal auditory meatus. The patient presented with tinnitus, unsteadiness, and difficulty with quick turns. On examination, he was positive for Romberg’s and Hitzelberger’s signs, indicating facial nerve compression deeper to the inner ear structures. Subsequent imaging confirmed indentation of the facial nerve [38]. Structures can also be compressed by the vessels around the meatus and the labyrinthine artery itself. An aneurysm of the labyrinthine artery within the IAM causes sudden onset of right facial paralysis and hyperacusis. In one case, a left-beating horizontal nystagmus was also noted; subsequent MRI revealed an aneurysm measuring 6 x 7 mm. Surgical clipping of this aneurysm resolved all the symptoms [39]. The structure of the anterior loop of the AICA (Anterior Inferior Cerebellar Artery) causes facial nerve irritation if it presses against the nerve within the meatus. This patient experienced painful hemifacial spasms that completely resolved after surgical separation of the AICA from the facial nerve.

## Conclusions

This review has examined the cranial nerve foramina of the posteriorcranial fossa. When their dimensions are compared with the sizes of their contents, we can estimate the amount of free space available; it is evident that lesions do not have to be large to cause impingement and significant clinical consequences. Therefore, a thorough knowledge of the foramina of the posterior cranial fossa is important for clinicians viewing images of this area or performing invasive procedures near this region. We hope this review provides a better understanding of the spatial anatomy and various pathologies of the posterior cranial fossa.
